# Functional classification and biochemical characterization of a novel rho class glutathione *S*-transferase in *Synechocystis* PCC 6803

**DOI:** 10.1016/j.fob.2014.11.006

**Published:** 2014-11-24

**Authors:** Tripti Pandey, Gaurav Chhetri, Ramesh Chinta, Bijay Kumar, Dev Bukhsh Singh, Timir Tripathi, Arvind Kumar Singh

**Affiliations:** a Department of Biochemistry, North-Eastern Hill University, Shillong 793022, India; b Department of Biotechnology, Institute of Biosciences and Biotechnology, Chhatrapati Shahu Ji Maharaj University, Kanpur 208024, India

**Keywords:** GST, glutathione *S*-transferase, GSH, reduced glutathione, DCA, dichloroacetate, CDNB, 1-chloro-2,4-dinitrobenzene, GSTZ, glutathione *S*-transferase zeta, Cyanobacteria, Detoxification, Glutathione, Dichloroacetate, Water pollutant, Bioremediation

## Abstract

We report a novel class of glutathione *S*-transferase (GST) from the model cyanobacterium *Synechocystis* PCC 6803 (sll1545) which catalyzes the detoxification of the water pollutant dichloroacetate and also shows strong glutathione-dependent peroxidase activity representing the classical activities of zeta and theta/alpha class respectively. Interestingly, sll1545 has very low sequence and structural similarity with these classes. This is the first report of dichloroacetate degradation activity by any bacterial GST. Based on these results we classify sll1545 to a novel GST class, rho. The present data also indicate potential biotechnological and industrial applications of cyanobacterial GST in dichloroacetate-polluted areas.

## Highlights

• A novel class of glutathione *S*-transferase (GST) is reported.
• This GST catalyzes dichloroacetate (DCA) degradation and hydroperoxide reactions.
• Functionally this GST is similar to zeta and theta/alpha classes but structurally very different.
• In contrast to other bacterial GSTs, this GST exists as a monomer in solution.
• First report of DCA degradation by any bacterial GST and has potential biotechnological applications.

## 1 Introduction

Dichloroacetate (DCA), a by-product of water chlorination and a metabolite of drug and industrial chemicals is known to cause nerve and liver damage [1–3]. It is a xenobiotic of interest to both environmental toxicologists and clinicians [3–5]. Its accumulation in groundwater and at certain Superfund sites is considered a potential health hazard. It has been shown that the chronic administration of DCA in mice induces hepatotoxicity and neoplasia [6]. In contrast, DCA stimulates the activity of pyruvate dehydrogenase enzyme complex of mitochondria, resulting in increased oxidation of blood glucose and lactate and thus can help in treatment of lactic acidosis [7–10]. A low dose of DCA is highly effective for treatment of congenital lactic acidosis [7]. Recent researches suggest that it may also enhance cellular energy metabolism and can help in cancer treatment [11,12]. Though human clinical studies showed that the elimination half-life of DCA increases with repeated doses of DCA. At higher concentrations in water bodies it can cause liver damage and tumors [6]. Hence detoxification of DCA containing wastewater is essential before its release [6].

Glutathione *S*-transferases (GSTs E.C. 2.5.1.18) are isozymes having central roles in the cellular detoxication of a diverse group of exogenous and endogenous harmful compounds [13]. GSTs catalyze the initial step in the formation of mercapturic acid derivatives of a wide range of foreign compounds [14]. They conjugate reduced glutathione (GSH) with compounds that contain an electrophilic center through the formation of a thioether bond between the sulphur atom of GSH and the substrate .In addition, these enzymes also carryout a range of other functions like nucleophilic aromatic substitution reactions, reversible Michael additions to α, β-unsaturated aldehydes and ketones, isomerizations, epoxide ring openings, and peroxidase reactions [13,15–17]. Despite low amino acid sequence identity, the global fold of GSTs is remarkably similar. Most cytosolic GSTs are dimeric and consists of two structurally distinct domains – the N-terminal thioredoxin like domain and a larger C-terminal all α-helical domain. The N-terminal domain contains the GSH binding site while the C-terminal domain is involved in substrate recognition. In some eukaryotes monomeric GSTs are also reported. GSTs have been divided into a number of classes – alpha, mu, pi, theta, sigma, beta, chi, omega, zeta, etc. – that is based on amino acid sequence similarity, immunological, kinetic, and tertiary/quaternary structural properties. Cytosolic GSTs within a class share >40% identity whereas there is only approximately 25% identity between classes [18].

It has been reported that zeta class GSTs are dimeric and can degrade DCA [19,20]. GSTZ are widely distributed in organisms including plants, fungi and eukaryotes [21–23]. Few structures of zeta class GSTs have been resolved by using X-ray crystallography [24,25]. Although these structures have the GST canonical fold, a number of structurally important differences exist. Presence of high concentrations of reduced glutathione suggests the occurrence as well as importance of GSH utilizing enzymes in cyanobacteria [26–29]. GSTs of Cyanobacteria have not been characterized in detail. We studied DCA degradation ability of a GST of the cyanobacterium *Synechocystis* PCC 6803. Database suggests that *Synechocystis* PCC 6803 encodes at least 3 GSTs. sll1545 is a *Synechocystis* GST that cannot be assigned to any class on the basis of sequence similarity. It shows only 21% sequence identity with zeta class and with alpha/theta classes of GSTs. Also most zeta class GSTs are ∼25 kDa protein, while sll1545 is encoded by 816 nts and is of about 30 kDa. In contrast to all previously reported dimeric bacterial GSTs, the recombinant sll1545 was found to be a monomeric protein of 30 kDa. Molecular modeling studies showed major differences between sll1545 and zeta class GSTs. Therefore in order to assign sll1545 its correct hierarchal position in GST superfamily we cloned sll1545 from *Synechocystis* PCC 6803, inserted into a His-tagged *Escherichia* *coli* prokaryotic expression system and studied its biochemical nature. The monomeric 30 kDa protein showed high specific activity and affinity for DCA as a substrate. Additionally, sll1545 shows peroxidase activity that is a signature of theta and alpha class of GSTs. Though, the structural and sequence similarity of sll1545 with these classes is very less. On the basis of these results we propose a novel rho class GST in *Synechocystis* PCC 6803 with potential for detoxification of DCA contaminated wastewater.

## 2 Materials

The molecular biology kits and Ni–NTA agarose were purchased from Qiagen, CA, USA. The dNTPs and enzymes were purchased from New England Biolabs, MA, USA. All other reagents and chemicals were purchased either from Sigma–Aldrich Chemical Company, St. Louis, MO, USA, or Sisco Research Laboratories, Mumbai, India and were of the highest purity available. Bacterial culture media was purchased from Himedia Laboratories, Mumbai, India.

### 2.1 Construction of a prokaryotic expression plasmid

The *Synechocystis* PCC 6803 was cultured in BG-11 medium. The genomic DNA was isolated using DNA isolation kit (Qiagen, USA). sll1545 gene was amplified using Phusion High-Fidelity DNA Polymerase (New England Biolabs, UK) using 5′-CGGGATCCATGCTTGAGCTT-3′ and 5′-AACTGCAGCTACTCAATGGTG-3′ as forward and reverse primers respectively. The restriction site consists of BamHI and PstI for the forward and reverse primers respectively. The PCR involved 30 cycles of denaturation at 98 °C for 20 s, annealing at 66 °C for 15 s followed by elongation at 72 °C for 15 s. This PCR product was digested with EcoRV and cloned into already EcoRV digested and purified pSK+ vector. The clone was verified by sequencing. After sequencing, the correctly cloned plasmid and pQE30 vector were both digested by BamHI and PstI restriction enzymes. The sll1545 gene fragment and the linear plasmid were recycled after agarose electrophoresis; connected by T4 DNA ligase to construct the recombinant expression plasmid pQE30-sll1545. The plasmid was transformed into *E. coli* DH5α competent cells and positive clones were screened. The correct pQE30-sll1545 clone was transformed into M15 competent cells for protein expression.

### 2.2 Induction of expression and purification of recombinant protein

Recombinant sll1545 was overexpressed in *E. coli* M15 cells and purified as follows. Single colony from transformed plates was inoculated in 5 mL of LB broth containing 100 μg/mL ampicillin and 50 μg/mL kanamycin. Cells were grown for 4–5 h at 37 °C with continuous shaking at 160 rpm. Next day a 400 mL LB broth flask containing above–mentioned antibiotics was inoculated with 1% (v/v) of overnight grown culture and incubated at 37 °C with shaking. Culture was grown until the OD_600_ reached 0.5–0.6. At this stage culture was induced with 1 mM IPTG. The culture was grown overnight at 23 °C. Next day culture was harvested and pelleted by centrifugation at 7000 rpm for 10 min at 4 °C. The pellet was then suspended in 1/50th culture volume of lysis buffer. The dissolved cells were lysed by sonication with pulse–rest cycle (60 cycles; 20 s pulse at 40% amplitude with 10 s interval after each pulse). The lysate was centrifuged at 12,000 rpm for 20 min at 4 °C and the supernatant was collected. All further steps were performed at 4 °C temperature. The supernatant was poured on Ni–NTA agarose matrix (3 mL) pre-treated with equilibration buffer (50 mM phosphate buffer pH 8.0 containing 300 mM NaCl) and was allowed to bind slowly. Non-specifically bound and contaminating proteins were removed by washing with equilibration buffer containing 50 mM imidazole. Recombinant protein was eluted with 10 mL of elution buffer (equilibration buffer containing 400 mM imidazole). The protein was dialyzed against 20 mM phosphate buffer pH 8.0 containing 150 mM NaCl, Protein concentration was determined by Bradford method using BSA as a standard.

### 2.3 Size exclusion chromatography

The determination of the native molecular weight of sll1545 was performed by size exclusion chromatography on a Superdex™ S-200 column (GE Healthcare Biosciences, USA). The calibration curve was made using the logarithm of the molecular mass of standard proteins vs. the elution volumes for conalbumin (75 kDa), ovalbumin (44 kDa), carbonic anhydrase (29 kDa), ribonuclease A (13.7 kDa) and aprotinin (6.5 kDa) (Gel Filtration Calibration Kit, GE Healthcare Biosciences).

### 2.4 Enzymatic activities

GST activity using GSH and CDNB as substrates was determined spectrophotometrically at 340 nm on the basis of the extinction coefficient for the product S–(2,4–dinitrophenyl) glutathione (*ε*_340_ nm = 9.6 mM^–1^ cm^–1^). The assay mixture (1 mL) comprisedsll1545 enzyme and 1 mM GSH in 100 mM phosphate buffer pH 8.0 containing 150 mM NaCl. The reaction was started by addition of 0.5 mM CDNB and reading was recorded for 60 s. During this period, the rate of reaction was linear with time. One unit of GST activity was defined as the conjugation of 1 μmol of CDNB with GSH per minute at 25 °C. The data was recorded with a Cary 50 Bio UV–Visible spectrophotometer at 25 °C.

The spectrometric method of Vogels and Van Der Drift was used to quantify glyoxylic acid formation using DCA as a substrate [30]. One mL of assay mixture contained 100 mM phosphate buffer (pH 7.4), 0.5 mM DCA and 1 mM GSH. Reaction was started by addition of DCA and 50 μL of trifluoroacetic acid was added to stop the reaction after 20 min of incubation at 37 °C. The reaction mixtures were placed on ice for 10 min and then centrifuged for 5 min to remove precipitated proteins. 850 μL of supernatant was transferred into a clean 5 mL microfuge tube and neutralized with 0.5 mL of 1 M NaOH. 300 μL of 800 mM phosphate buffer (pH 6.8) and 400 μL of phenylhydrazine HCl solution (100 mg in 15 ml of water) were added to each tube and the contents were mixed properly. The mixtures were kept at 25 °C for 10 min and then placed on ice for 5 min. 1 mL of chilled, concentrated HCl and 400 μL of potassium ferricyanide solution (500 mg in 15 mL of water) were added to each sample. The samples were thoroughly mixed and then kept at 25 °C for 15 min; the absorbance was measured at 535 nm against water. Blank reaction lacked DCA. Glyoxylic acid concentrations were quantified with a standard curve prepared under the same conditions. Standard curve was made using 4–32 nmol of glyoxylic acid in a final volume of 1 mL. The kinetics of the glutathione-dependent enzymatic oxygenation of DCA was studied as described above. Reaction mixtures contained 0.25 μg of purified protein and 0.05–1 mM DCA when the km and Vmax for the oxygenation of DCA at a fixed glutathione concentration of 1 mM were studied. To measure the km for glutathione, a fixed concentration of 0.5 mM DCA was used, and glutathione concentrations ranged from 0.05 to 1 mM. The km and Vmax were calculated using Prism 3.0 (Graphpad Prism Software, San Diego, USA).

The pH optimum for DCA conjugation activity was determined for DCA conjugation activity using citrate/glycine/hepes (CGH) buffer of various pH values. Purified sll1545 was incubated at 30 °C for 30 min in CGH buffer of pH values ranging from 5.5 to 9.5. Conjugation activity was determined as described above. Three replicate experiments were conducted and the background values were subtracted for all experiments. For temperature dependent activity, the protein was incubated from 20 °C to 80 °C for 10 min and then activity was taken at the same temperature.

### 2.5 Structural superimposition and phylogeny

MatchMakermodule of UCSF Chimera 1.9 was used for structural alignment of 3D structure of human GST (PDB ID: 1FW1) with modeled 3D structure of sll1545. It superimposes structures pairwise by first aligning their sequences and then fitting the α-carbons of residues in the same columns of the sequence alignment.

BLAST search was carried out to trace the sequences that share maximum similarity and homology with the query protein (sll1545). Fourteen sequences were selected from BLAST hit on the basis of identity, positive and E-value score. Multiple sequence alignment for all fifteen sequences of GSTs from different species was performed using ClustalW2. ClustalW2 was also used to generate phylogenetic tree for the aligned sequences.

## 3 Results

**Fig. 1:**
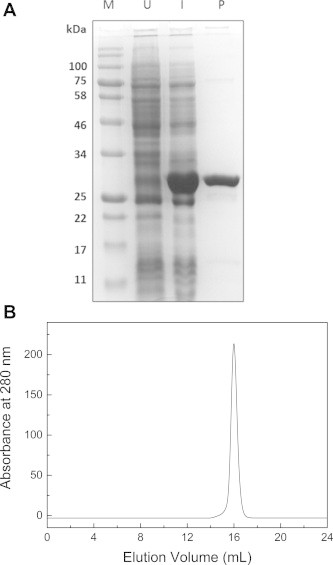
Overexpression of sll1545 in *E. coli* and purification of the recombinant protein on Ni–NTA agarose. (A) SDS–PAGE analysis of cell lysate showing overexpression of sll1545 and the purified protein. Lanes 1–4 represent molecular weight markers, supernatant of un-induced culture lysate, supernatant of induced culture lysate and purified protein, respectively. (B) Molecular weight and subunit structure of sll1545. SEC profile of sll1545 on Superdex™ 200 10/300 GL column at pH 8.0 and 25 °C.

PCR amplification of sll1545 gene generated a product of ∼800 bp that was subsequently cloned in pSK+ vector. It was further sub-cloned in pQE30 vector for protein expression. The resulting plasmid was transformed in *E coli* M15 cells and the protein was expressed using T5 promoter and IPTG system. The expression of the protein was moderate and it was found to be in the soluble fraction. The protein was then purified to homogeneity using Ni–NTA matrix (Fig. 1A). The oligomeric structure of the protein was determined using Superdex S-200™ column SEC. The protein eluted at an elution volume of 16.0 mL that corresponds to about 30 kDa when compared with the molecular weight standards (Fig. 1B). This indicates that under native conditions sll1545 exists in a monomeric state. This was further confirmed using native-PAGE where a band of 30 kDa was observed (data not shown).

**Fig. 2:**
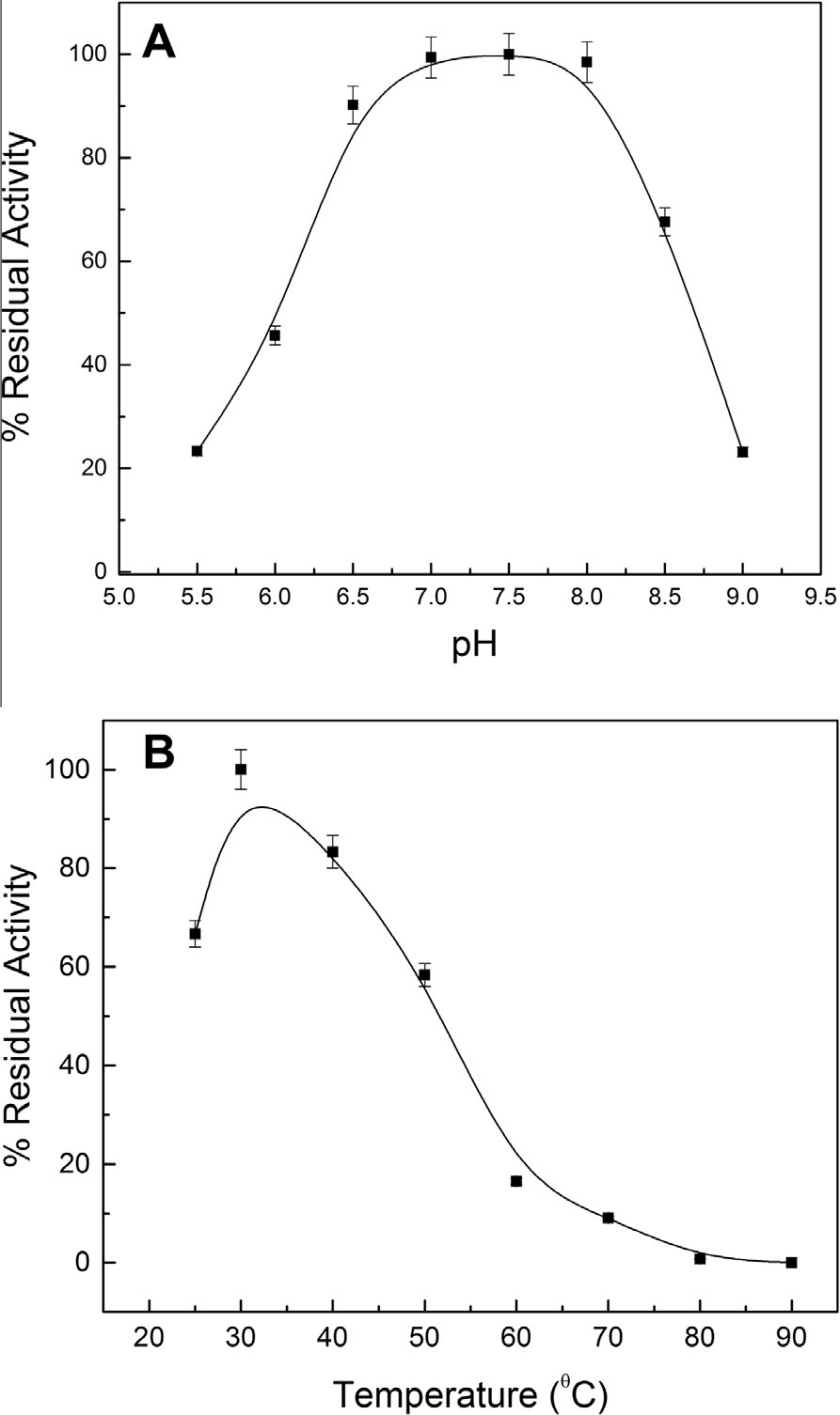
Effect of pH and temperature on the enzymatic activity of sll1545. (A) Effect of pH on the catalytic activity of sll1545. (B) Effect of temperature on the activity of sll1545.

The purified recombinant protein was kinetically examined with CDNB as a model substrate for GST catalyzed reactions. While protein exhibited moderate GSH-transferase activity with CDNB, it showed strong catalytic activity with DCA having kcat of 82.17 s^−1^ and high enzymatic affinity with km value around 0.143 mM. The kinetic parameters with DCA and GSH are given in Table 1. The pH optimum of sll1545 with DCA as substrate was found to be 7.5. At pH value below 7.0 and above 8.5 the activity decreased substantially (Fig. 2A). Temperature dependent studies revealed maximum activity at 30 °C which reduced to approximately 60% at 50 °C. The enzyme showed drastic decrease in activity at temperatures above 60 °C (Fig. 2B).

sll1545 was used as query in protein BLAST tool to find out the closely related sequences. BLAST search was performed against non-redundant database of protein. Total 100 BLAST hits were obtained on the query sequence (sll1545). Multiple sequence analysis between 15 GSTs and sll1545 was performed. The results showed various conserved and non-conserved regions. Phylogenetic tree was been created to trace out their evolutionary relationship and direction of evolution.

## 4 Discussion

**Fig. 3:**
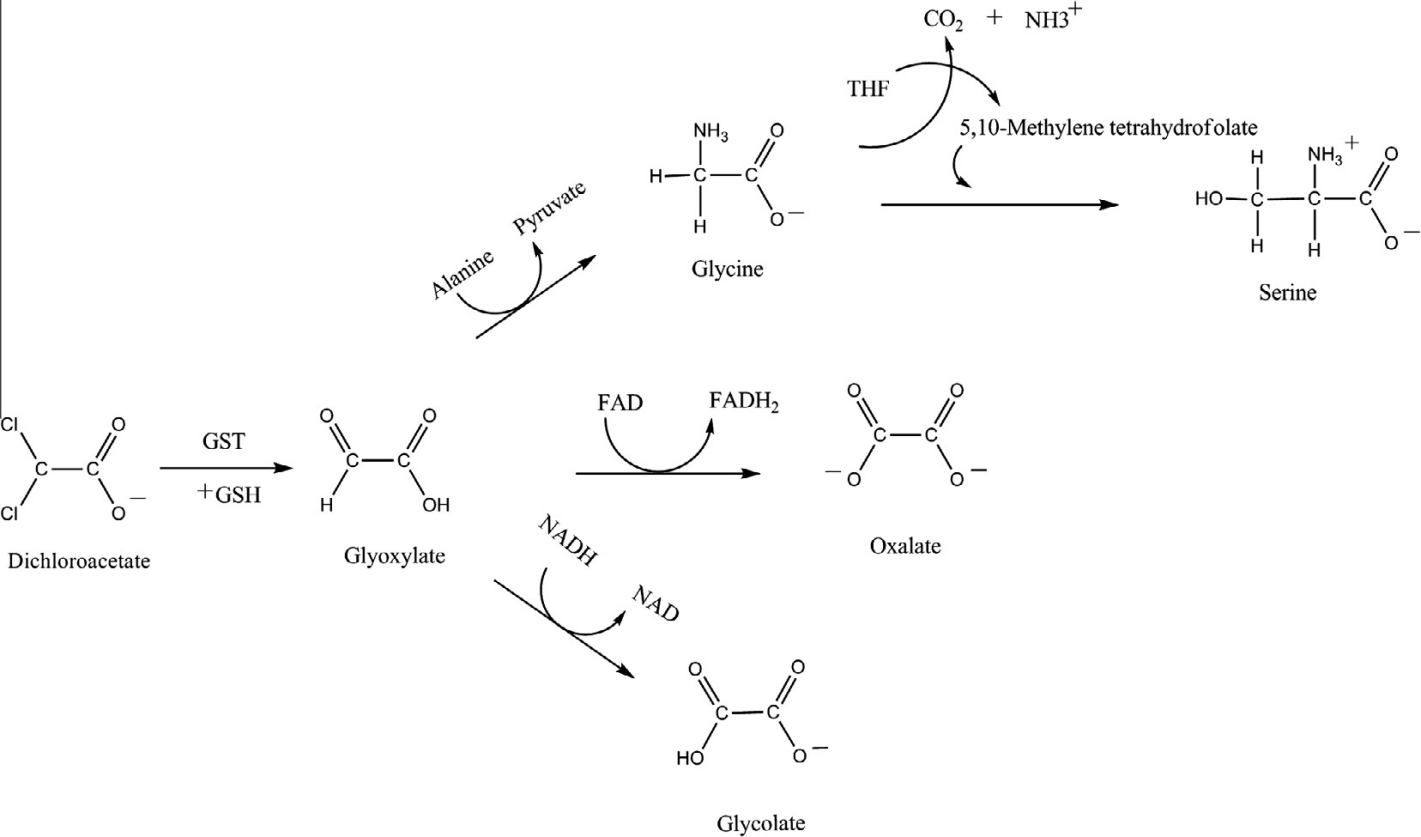
Fate of DCA and metabolic pathways for degradation of glyoxylate.

The primary metabolic pathway for DCA involves oxidative dechlorination to form glyoxylate. This reaction, once thought to be microsomal cytochrome P-450 mediated, has now been shown to be NADPH and GSH dependent that occurs predominantly in the cytosol. Glyoxylate can enter intermediary metabolism and the carbon atoms originally present in DCA can get incorporated into endogenous proteins and other biomolecules. Glyoxylate may be routed through several different pathways as sown in Fig. 3. Transamination by peroxisomal alanine-glyoxylate transaminase forms glycine that can be either incorporated into proteins or used in the synthesis of the amino acid serine or can be degraded releasing CO_2_. Conversion of glyoxylate to oxalate also occurs via lactate dehydrogenase utilizing FAD. Glyoxylate can also be converted to glycolate by glyoxylate reductase.

**Fig. 4:**
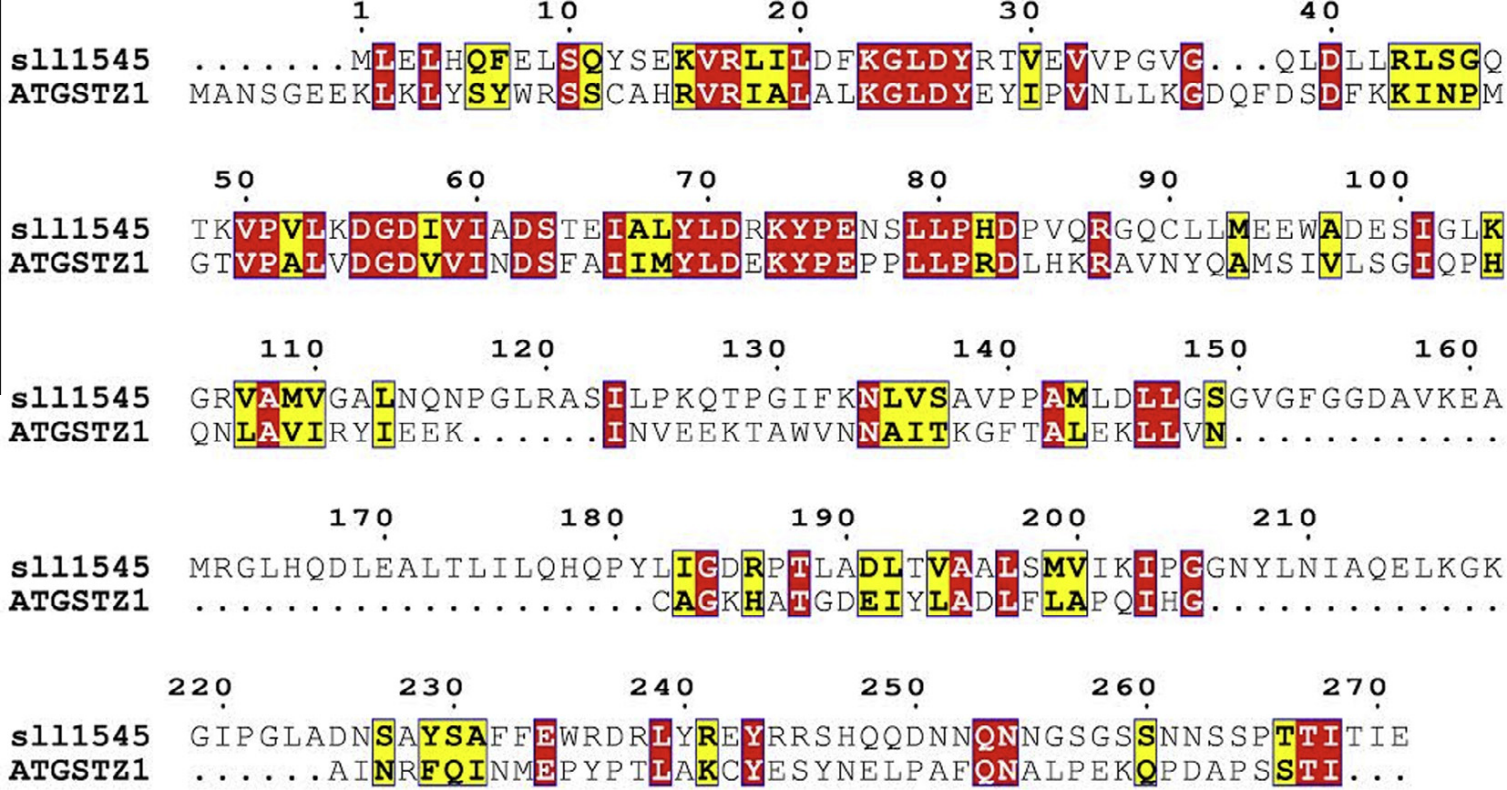
Multiple amino acid sequence alignment. *Synechocystis* PCC 6803 sll1545 (P74665) and *Arabidiopsis thaliana* zeta GST (Q9ZVQ3) were aligned using ESpript 3.0 software utilizing the clustalW algorithm. Similar residues are shown in yellow boxes, red boxes represent identical amino acid residues while residues having different property are without boxes. (For interpretation of the references to color in this figure legend, the reader is referred to the web version of this article.)

**Fig. 5:**
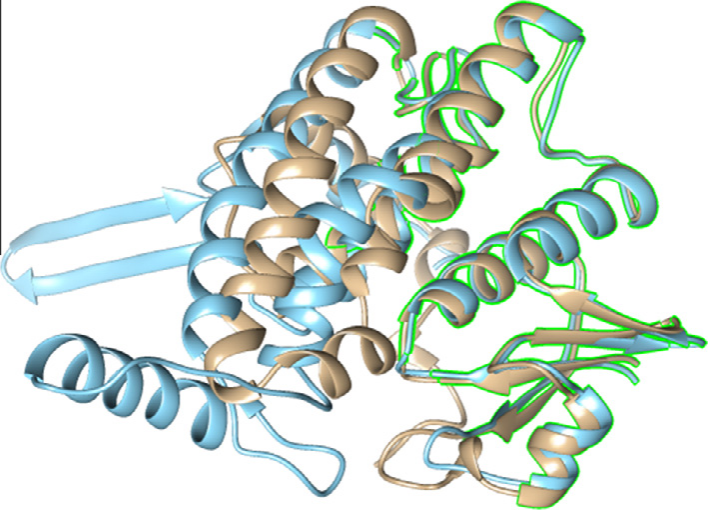
Superposition of the molecular model of sll1545 with a zeta class GST. Homology modeling of sll1545 was performed using swissmodel. The model was superimposed on the structure of human zeta class GST (PDB ID: 1FW1). Golden and blue color represents 1FW1 and the sll1545 respectively. Green line-represents structurally aligned or superimposed residues in both. The structures were visualized using UCSF Chimera 1.9. (For interpretation of the references to color in this figure legend, the reader is referred to the web version of this article.)

Zeta class GST appears to be similar to maleylacetoacetateisomerase (MAAI), the enzyme in the pathway for tyrosine catabolism that converts the cis double bond in maleylacetoacetate to the trans double bond in fumarylacetoacetate. This reaction requires GSH as a cofactor. GSTZ/MAAI has an active site geometry that is highly conserved across species and is sufficiently flexible to participate in cis/trans isomerization reactions and dehalogenation of diverse molecules like DCA and pentachlorophenol. Our results suggest that sll1545 is a distantly related orthologue of zeta class GST. Functionally it is similar to GSTZ, but has very different structure and sequence. Only 20% amino acid sequence similarity exists between GSTZ and sll1545 (Fig. 4) suggesting that sll1545can be of a different class than zeta. The protein exists as a monomer in solution and catalyzes the dehalogenation of DCA to glyoxylate. Thorough kinetic examination has not been performed with GSTZ, thus we studied the kinetic parameters of sll1545 in detail (Table 1). The km for DCA is very low indicating its high affinity for the enzyme. The specificity constant kcat/km value is a measure of the catalytic efficiency and represents the catalytic capacity at low substrate concentrations. Our results show very high catalytic efficiency of sll1545 with DCA as substrate. The molecular model of sll1545 was superimposed on the human zeta class GST (PDB ID: 1FW1). The structural alignment shows major deviations in the C-terminal and middle region (Fig. 5). Alignment score between human GST (1FW1, chain A) and sll1545 (model of chain A) was 284.6. RMSD was calculated using one point per residue Cα in amino acids. The two structures generated a RMSD of 0.942 Å over 109 alpha carbon pairs. RMSD calculates per-column spatial variation among the associated structures and is only calculated for columns with at least two associated structure residues. Structurally aligned residues of human GST with modeled 3D structure of sll1545 are denoted by pink boxes and labels that correspond to these locations are highlighted on the superimposition diagram by green color (Supplementary Fig. S1). Sequence composition and structural similarity statistics indicates that zeta class GST is not much similar to GST of *Synechocystis* sp. PCC 6803*.* Superposition of these two structures shows that they are structurally conserved at N-terminal region as compared to C-terminal. Amino acid residues (Lys5-Ala107) of human GST are well structurally aligned with the residue (Met1-Asp98) of modeled structure from sll1545 at N-terminal region. While both contains two interruptions in structural concreteness with a small insert at C-terminal region. Another, small structurally conserved block is present at C-terminal region which superimposes the amino acid residues of human GST from Tyr153 to Val170 with the residue Tyr181 to Ser198 of modeled structure from sll1545. Unpublished data from our lab suggests strong GSH dependent peroxidase activity of sll1545. This type of activity is a signature of alpha and theta classes of GSTs. Interestingly sll1545 shows very less similarity (∼20%) with these classes. The structural alignments also show major deviation between sll1545 and alpha/theta classes. Based on these results we propose sll1545 to be of a novel GST class and name it as rho class.

**Fig. 6:**
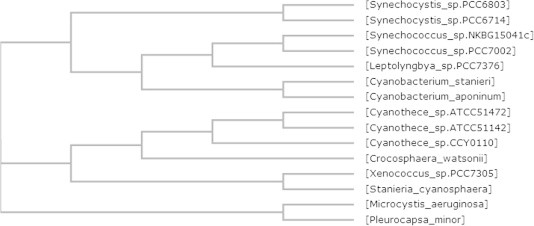
Phylogenetic tree of GST sequences from different species that are similar to GST-sll1545 of *Synechocystis* PCC 6803.

Detail bioinformatics analysis was performed to understand the phylogeny and sequence conservation of sll1545. The main motivation behind BLAST analysis was to find out sequences similar to sll1545. Putative conserved N- and C-terminal domains of GSTs were detected in sll1545 suggesting that sll1545 belong to GST super family. Fourteen closely related sequences were taken for building phylogenetic tree (Fig. 6). Descriptions such as query coverage; *E* value; identity and accession details of closely related sequences are listed in Supplementary Table 1. sll1545 showed maximum identity (93%) and similarity (95%) with GST of *Synechocystis* sp. PCC 6714. Sequence identity for remaining sequences ranges between 62% and 69%. GST of *Pleurocapsa minor* was found to have 69% identity and 80% similarity with sll1545. Multiple sequence alignment (MSA) for all15 sequences of GSTs from different species indicates a high degree of similarity among GST sequences though few mutational regions throughout the entire length were observed. For better understanding of similarity, divergence, evolutionary relationship and direction of evolution a phylogenetic tree comprising all these sequences of GST were constructed using ClustalW2.sll1545 and *Synechocystis* sp. PCC 6714GST are very closely related to each other during course of evolutions they fall under same clade. Similarly, two species *Synechococcus* sp. NKBG15041c and *Synechococcus* sp. PCC 7002 are closely related. GST of *Cyanothece* sp. CCY0110 is distinct and distantly related with the other two *Cyanothece* species. GST of *Synechocystis*, *Leptolyngbya* and other cyanobacterial species are evolutionary very close as compared to GST of *Cyanothece*, *Crocosphaera*, *Xenococcus*, *Stanieria*, *Microcystis* and *Pleurocapsa* species that seem to be evolve from a common ancestor.

## 5 Conclusion

The data presented here suggest the occurrence of a novel GST class in *Synechocystis* PCC 6803 that is functionally similar to but structurally different from zeta, theta and alpha classes of GSTs. The high affinity of purified GST to DCA and its ability to metabolize it indicates its potential in detoxification of DCA loaded industrial effluents. Detailed characterization of DCA detoxification by genetically engineered *Synechocystis* PCC 6803 strain can be extremely useful for developing predictive models of DCA bioremediation. Further studies are in progress and we will address this issue in future.

## Conflict of interest

The authors declare that there are no conflicts of interests.

## Figures and Tables

**Table 1:** Kinetic parameters of sll1545 with DCA as substrate. Enzymatic activities were measured at various concentrations of GSH and DCA as described in Experimental section. Kinetic constants are based on three independent experiments for each measurement. Results are means ± S.D. of three independent measurements as described in Section 2.

	GSH	DCA
km (mM)	1.50 ± 0.16	0.14 ± 0.02
Vmax (μmole min^−1^)	23.22 ± 0.07	11.75 ± 0.02
Specific activity (nmol min^−1^ mg^−1^)	56.45 ± 1.23	237.70 ± 1.03
kcat (s^−1^)	15.48 ± 0.48	82.17 ± 0.23
kcat/km (s^−1^ mM^−1^)	10.32 ± 0.66	574.59 ± 0.99

## Appendix A Supplementary data

Supplementary data 1 This supplementary data consists of Fig. S1 and Table 1.
