# Supplementary material for: Functional classification and biochemical characterization of a novel rho class glutathione S-transferase in Synechocystis PCC 6803
Source: FEBS Open Bio. 2014 Nov 24;5:1–7. doi: 10.1016/j.fob.2014.11.006 (PMC4309839; doi:10.1016/j.fob.2014.11.006)
Supplement: Supplementary data 1 — This supplementary data consists of Fig. S1 and Table 1. [file mmc1.doc]

**Supplementary Figure S1:**

**Structural superimposition:** Structurally aligned residues of human GST (1FW1: PBD) with modeled 3D structure of sll1545, are denoted by pink boxes and labels that correspond to these locations are highlighted on the superimposition diagram by green color (Supplementary FigureS1)

**
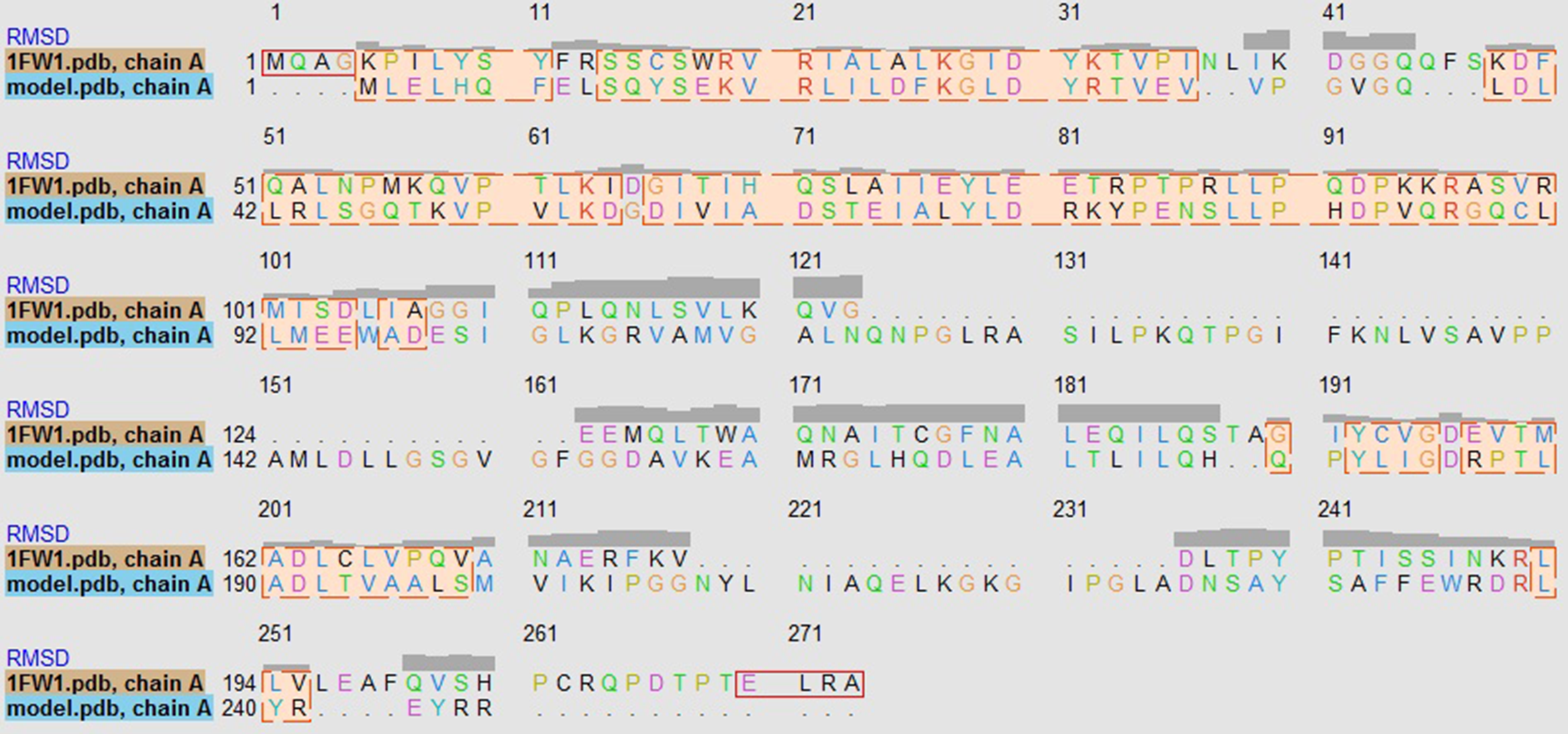
**

**Supplementary Table 1:** Description of sequences closely related to GST sll1545 of *Synechocystis* sp. PCC 6803.

| **S. No.** | **Sequences producing significant alignments** | **Total score** | **Positives** | **E value** | **Identity** | **Accession** |
| --- | --- | --- | --- | --- | --- | --- |
|  | glutathione S-transferase [Synechocystis sp. PCC 6714], 268 AA | 498 | 95% | 5e-176 | 93% | WP_028948484.1 |
|  | glutathione S-transferase [Synechococcus sp. NKBG15041c], 266 AA | 385 | 81% | 1e-131 | 67% | WP_024545657.1 |
|  | glutathione S-transferase [Pleurocapsa minor], 263 AA | 383 | 80% | 8e-131 | 69% | WP_015143783.1 |
|  | glutathione S-transferase [Synechococcus sp. PCC 7002], 266 AA | 378 | 80% | 8e-129 | 65% | WP_012305757.1 |
|  | glutathione S-transferase [Crocosphaera watsonii], 263 AA | 377 | 80% | 2e-128 | 66% | WP_007308115.1 |
|  | glutathione S-transferase [Cyanothece sp. ATCC 51472], 263AA | 375 | 79% | 1e-127 | 66% | WP_009543034.1 |
|  | glutathione S-transferase [Cyanothece sp. ATCC 51142], 269 AA | 375 | 79% | 2e-127 | 66% | WP_012361303.1 |
|  | glutathione S-transferase [Cyanothece sp. CCY0110], 263 AA | 372 | 78% | 1e-126 | 65% | WP_008275980.1 |
|  | glutathione S-transferase [Leptolyngbya sp. PCC 7376], 266 AA | 372 | 80% | 1e-126 | 64% | WP_015133952.1 |
|  | glutathione S-transferase [Xenococcus sp. PCC 7305], 267 AA | 367 | 81% | 2e-124 | 64% | WP_006511001.1 |
|  | glutathione S-transferase [Microcystis aeruginosa], 265 AA | 353 | 77% | 6e-119 | 62% | WP_002758970.1 |
|  | Glutathione S-transferase domain protein [Stanieria cyanosphaera], 264 AA | 352 | 81% | 1e-118 | 65% | WP_015192116.1 |
|  | glutathione S-transferase [Cyanobacterium stanieri], 263 AA | 351 | 77% | 3e-118 | 65% | WP_015223670.1 |
|  | glutathione S-transferase [Cyanobacterium aponinum], 270 AA | 350 | 78% | 8e-118 | 65% | WP_015219661.1 |
